# Double burden of malnutrition in thin children and adolescents: low weight does not protect against cardiometabolic risk

**DOI:** 10.1038/s41430-021-00963-w

**Published:** 2021-07-06

**Authors:** Jonathan C. K. Wells

**Affiliations:** Childhood Nutrition Research Centre, Population Policy and Practice Research and Teaching Department, UCL Great Ormond Street Institute of Child Health, London, UK

**Keywords:** Malnutrition, Biomarkers

If the global epidemic of non-communicable disease (NCD) has a single defining marker, it is seemingly the relentless global increase in body mass index (BMI). At the population level, an increased prevalence of people with high BMI tends to indicate not only higher levels of harmful body fat, but also exposure to other NCD risk factors, such as lipogenenic diets and sedentary behaviour. Within recent decades, the global prevalence of low BMI in adults has steadily fallen, whereas that of high BMI, reflecting obesity, has systematically increased in most countries. Increasingly, rising BMI is observed in younger age groups too, though rates of child undernutrition remain higher than in adults [1].

**Fig. 1:**
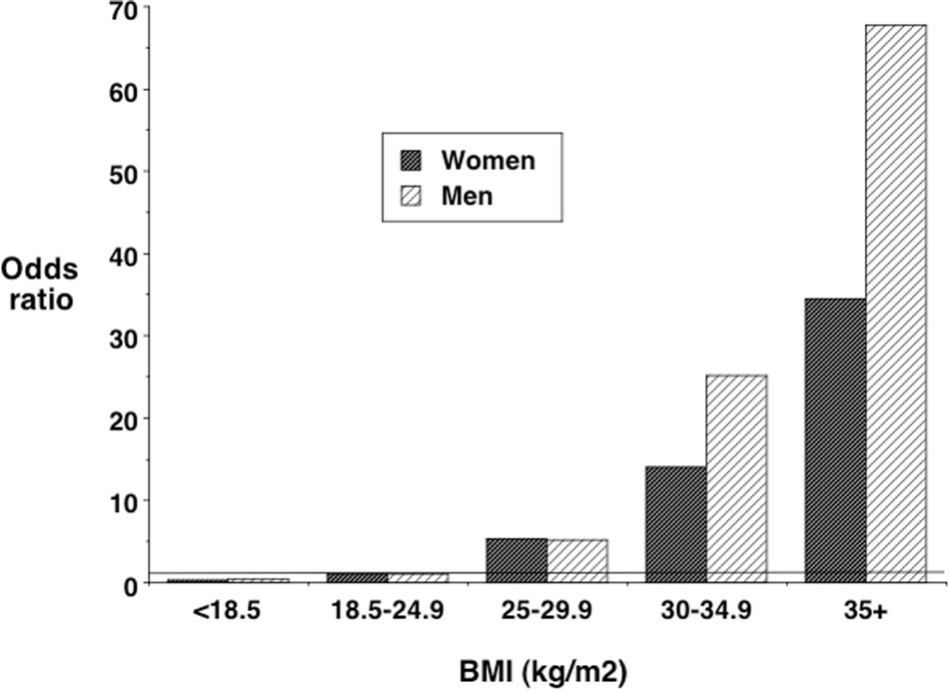
Odds ratios for the metabolic syndrome in men and women aged >20 years in the Third US National Health and Nutrition Examination Survey, according to body mass index (BMI). Data from ref. [2].

So well established is the epidemiological link between high BMI and NCD risk that it might seem obvious that low BMI is strongly protective against cardiometabolic risk. For example, data from the third National Health and Nutrition Examination Survey in the US (conducted 1988–1994) highlighted the exponential increase in risk of the metabolic syndrome in adults >20 years in association with BMI in this time period (Fig. 1) [2]. Those with low BMI are expected to suffer primarily from nutritional problems such as dietary deficiencies and impaired somatic and skeletal growth. From a life-course perspective, there is growing evidence that the association of high BMI with NCD risk in adult life is exacerbated among those who also experienced undernutrition in early life [3]; but if children do not have excess weight, surely they have a healthy cardiometabolic profile?

New data from India published in this issue send a shockwave through this assumption. Sachdev and colleagues analysed data on children and adolescents from the large Comprehensive National Nutrition Survey (2016–2018) [4]. Whilst overweight and obesity are increasingly evident in the Indian paediatric population, a relatively high proportion remain categorised as underweight according to the anthropometric criteria of the World Health Organisation. Strikingly, the study found that even in the undernourished groups, a high proportion of the children and adolescents had elevated cardiometabolic risk markers. In these young individuals, thinness has offered no protection against metabolic perturbations that are conventionally associated with excess adiposity. The authors interpret this as the consequence of ‘metabolic obesity’ in the absence of high BMI.

Defined in anthropometric terms, thin children cannot have high levels of body fat in an absolute sense. So where has their NCD risk come from, and how is the concept of obesity relevant? There are several possible pathways.

First, dietary quantity and quality require differentiation. Highly processed foods are not only problematic for nutritional health (being deficient in protein, fibre and micronutrients, and high in sugar and fat content), but are also relatively cheap [5]. Their aggressive marketing has already attracted major concern, specifically in relation to school children [6]. Studies in Indian slum-dwelling adults have already shown that unhealthy diets can raise biochemical markers of NCD risk in the absence of high BMI [7]. Therefore, underweight children may likewise be increasingly consuming diets that harm metabolic health.

Second, inadequate physical activity could be contributing, even if not through the pathway of promoting high BMI. As well as sedentary behaviour, unhealthy sleep patterns have also been shown to impair metabolic homoeostasis.

Third, even if overall fatness is not high, its regional anatomical distribution may be unhealthy. According to data in adults, fat distribution has a complex association with cardiometabolic health, whereby central abdominal fat promotes NCD risk, while peripheral fat may protect against it [8]. A more central fat distribution may therefore generate the metabolic signature of obesity, even at lower levels of body weight.

Fourth, there is increasing evidence that the association of nutritional status and NCD risk is mediated by the profile of the gut microbiome. Independently, both undernutrition and overweight have been associated with dysbiosis, through the findings show heterogeneity [3]. Recent work has shown that changing the microbiotic content of food may help resolve undernutrition and promote healthier growth [9]; potentially, this approach may also benefit long-term cardiometabolic health too.

Fifth, broader aspects of the environment may also contribute. For example, the role of air pollution in both stunting and NCD risk is increasingly recognised [10], and in countries such as India where biomass cooking is still practised, many young children from poorer households may be especially exposed within the home.

**Fig. 2:**
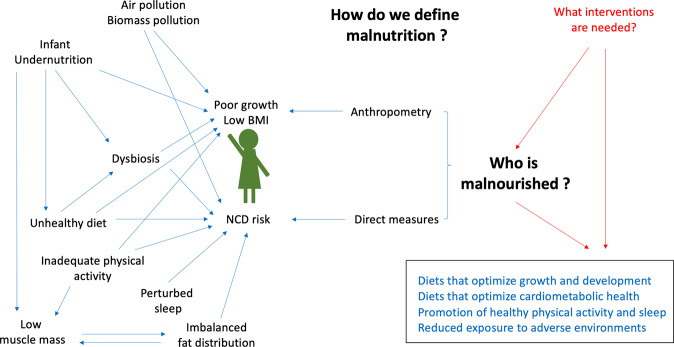
Multiple factors may contribute to malnutrition in its broad sense in children who are not overtly overweight. Given new evidence on the co-occurrence of markers of undernutrition and cardiometabolic risk in individual children, new approaches are needed to evaluate the double burden of malnutrition that go beyond anthropometry.

All of these pathways, as well as others not discussed here due to lack of space, may be relevant, as illustrated in the schematic diagram in Fig. 2. The sobering message is that NCD risk appears already to be increasing in young Indian children and adolescents whether or not they have high BMI. This prompts a number of important issues in relation to monitoring nutritional health and designing interventions to improve it.

First, tracking the early manifestation of malnutrition can no longer be based on anthropometric criteria alone, if NCD risk is to be monitored. This requires major rethink of an approach that is currently the backbone of public health in the paediatric population. Second, much greater effort must be invested in identifying the determinants of NCD risk that do not operate via high BMI. It has long been problematic that much greater use has been made of biotechnology to probe body composition and metabolic profile in those overweight, compared to those underweight; this imbalance requires urgent correction. Third, the study adds to growing evidence challenging the notion that there are discrete populations who require interventions that essentially either promote, or constrain, nutritional intake in order to reduce undernutrition or overnutrition respectively, and thereby promote nutritional health. Instead, the thin children with elevated NCD risk described in this population require interventions that simultaneously improve the supply of some nutrients while also reducing their exposure to other unhealthy nutrients, behaviours and environmental factors. Overall, as Sachdev and colleagues highlight their study provides crucial evidence of the challenges posed by the double burden of malnutrition at the individual level, and of how we need to change our approach to the assessment of nutritional status in order to address it.
